# Deciphering the Roles of BamB and Its Interaction with BamA in Outer Membrane Biogenesis, T3SS Expression and Virulence in *Salmonella*

**DOI:** 10.1371/journal.pone.0046050

**Published:** 2012-11-05

**Authors:** Fatémeh Namdari, Genaro Alejandro Hurtado-Escobar, Nadia Abed, Jérôme Trotereau, Yann Fardini, Etienne Giraud, Philippe Velge, Isabelle Virlogeux-Payant

**Affiliations:** 1 INRA, UMR1282 Infectiologie et Santé Publique, Nouzilly, France; 2 Université François Rabelais de Tours, UMR1282 Infectiologie et Santé Publique, Tours, France; Arizona State University, United States of America

## Abstract

The folding and insertion of β-barrel proteins in the outer membrane of Gram-negative bacteria is mediated by the BAM complex, which is composed of the outer membrane protein BamA and four lipoproteins BamB to BamE. In *Escherichia coli* and/or *Salmonella,* the BamB lipoprotein is involved in (i) β-barrel protein assembly in the outer membrane, (ii) outer membrane permeability to antibiotics, (iii) the control of the expression of T3SS which are major virulence factors and (iv) the virulence of *Salmonella*. In *E. coli,* this protein has been shown to interact directly with BamA. In this study, we investigated the structure-function relationship of BamB in order to assess whether the roles of BamB in these phenotypes were inter-related and whether they require the interaction of BamB with BamA. For this purpose, recombinant plasmids harbouring point mutations in *bamB* were introduced in a Δ*Salmonella bamB* mutant. We demonstrated that the residues L173, L175 and R176 are crucial for all the roles of BamB and for the interaction of BamB with BamA. Moreover, the results obtained with a D229A BamB variant, which is unable to immunoprecipitate BamA, suggest that the interaction of BamB with BamA is not absolutely necessary for BamB function in outer-membrane protein assembly, T3SS expression and virulence. Finally, we showed that the virulence defect of the Δ*bamB* mutant is not related to its increased susceptibility to antimicrobials, as the D227A BamB variant fully restored the virulence of the mutant while having a similar antibiotic susceptibility to the Δ*bamB* strain. Overall, this study demonstrates that the different roles of BamB are not all inter-related and that L173, L175 and R176 amino-acids are privileged sites for the design of BamB inhibitors that could be used as alternative therapeutics to antibiotics, at least against *Salmonella.*

## Introduction

The outer membrane proteins of Gram-negative bacteria are synthesized in the cytoplasm and have to cross the inner membrane before being assembled into a correctly folded state in the outer membrane. The β-barrel assembly machinery (BAM) is an outer membrane complex responsible for recognition, folding and insertion of outer-membrane β-barrel proteins in the outer membrane (For a review, see [1]. In *Enterobacteriaceae*, such as *Escherichia coli* and *Salmonella*, the BAM complex is composed of the essential outer membrane protein, BamA (formerly YaeT), and four lipoproteins, BamB to BamE (formerly YfgL, NlpB, YfiO and SmpA respectively) [2], [3], but this composition can differ in other bacteria [4], [5]. BamA is conserved among bacteria, and homologues of the BAM complex are found in chloroplasts and mitochondria where they also assemble β-barrel proteins [6], [7], [8]. This essential protein interacts with the other Bam proteins via five POTRA (polypeptide transport-associated) domains present in its N-terminal periplasmic part. BamC, BamD and BamE proteins form a subcomplex that interacts with BamA *via* POTRA 5 while BamB/BamA interaction involves POTRA 2, 3, 4 and 5 domains [9]. The BAM complex has been shown to be involved in the assembly of integral outer membrane proteins (OMP) such as OmpA, LamB and the fimbrial usher protein FimD [10], [11]. This complex is also required for autotransporter biogenesis in several organisms [12]–[15]. Moreover, mutants unable to synthesize one or several Bam proteins have been shown to be more susceptible to different antibiotics including vancomycin, thus suggesting increased outer membrane permeability due to a defect in outer membrane integrity [11], [16].

In the BAM complex, BamB is not essential for bacterial viability but is required to maintain a wild-type level of OMP and correct outer-membrane permeability of the bacterium to antibiotics [11], [17], [18]. It is also involved in the assembly and secretion of some autotransporters, such as the extracellular serine protease P (EspP) autotransporter of *E. coli* O157 H7 [15], [19]. Moreover, previous studies in our laboratory have shown that the *bamB* gene plays an important role in the virulence of *Salmonella enterica* subsp. e*nterica* ser. Enteritidis (*S.* Enteritidis) in mice and in one-day-old chicks [18], [20]. The virulence defect of a *bamB* mutant may, at least in part, be related to the down-regulated transcription of most flagellar, T3SS-1- and T3SS-2- related genes, which encode major virulence factors [18]. A role of BamB in the invasion capability of an adherent invasive *E. coli* strain has also been described [21]. Recent studies have provided new data on the BamB protein and its putative role in the BAM complex. By reconstructing the BAM complex in proteoliposomes, Hagan *et al.* [22], [23] confirmed that this protein, although not essential in *E. coli*, is important for the assembly of OMPs, and they demonstrated that its absence greatly impaired the activity of the two essential proteins of the complex, BamA and BamD. Moreover, Ieva *et al.* [19] showed that BamB (and BamD) remained bound to the C-terminal β-domain of the EspP autotransporter longer than BamA, suggesting that this lipoprotein plays a direct role in the assembly of β-barrel proteins at a later stage than BamA. Structural studies show that BamB is an eight-bladed β-propeller protein that contains WD40 repeat-like domains which are usually found in scaffolding proteins [24], [25], [26]. Some specific residues of BamB (L173, L175, R176, D227 and D229), which are important for its interaction with BamA and for outer-membrane permeability, have already been identified in *E. coli* [16]. These residues are conserved in *Salmonella.* Although they are distant in the amino-acid sequence, these residues form a continuous solvent-exposed surface on the β-propeller [25].

These data show that BamB is involved in different bacterial functions: β-barrel protein assembly, outer membrane permeability to antibiotics, T3SS expression and virulence. However, despite an increasing number of papers on this protein, it is currently not known whether the functions of BamB are inter-related or whether all of them require the interaction of BamB with the BAM complex. In order to answer these questions, we characterized, in *Salmonella,* BamB point mutants previously shown to alter or not the outer membrane permeability and the interaction of BamB with BamA in *E. coli* [16]. We chose to work on the BamB point mutants showing the least (L173S and R176A BamB variants) and the most altered (L173S,L175S,R176A triple-mutated BamB) phenotypes tested in *E. coli*. We also tested D227A and D229A BamB variants that were only impaired in their interaction with BamA in *E. coli*. The impact of these mutations on all the phenotypes previously described for the BamB protein, i.e. its interaction with BamA and its role in OMP biogenesis, outer membrane permeability, T3SS expression/functionality and virulence was investigated. Our results demonstrate that the different roles of BamB are not all inter-related and suggest that the interaction of BamB with BamA is not absolutely necessary in *Salmonella* for correct OMP biogenesis, optimal T3SS expression and virulence in mice.

## Results

### Influence in *Salmonella* of amino-acid substitutions in BamB protein on its interaction with BamA

**Figure 1:**
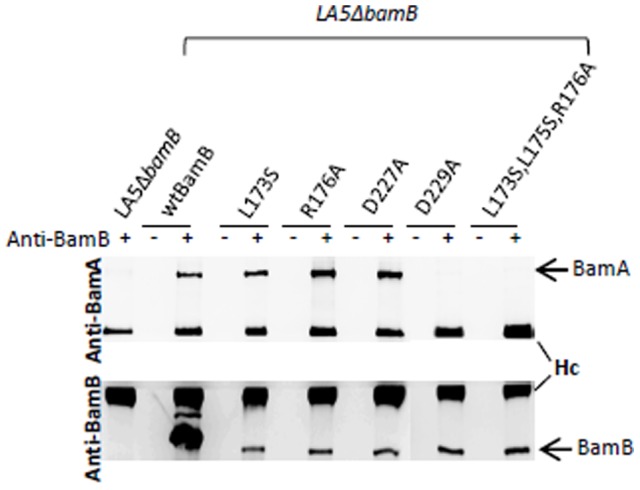
Effect of amino-acid substitutions in BamB proteins on the interaction of BamB with BamA. Co-immunoprecipitation of BamA with histidine-tagged BamB proteins was performed. Whole-cell lysates of the *S.* Enteritidis LA5Δ*bamB* mutant or the LA5Δ*bamB* mutant expressing wild-type BamB protein, BamB with the single L173S, R176A, D227A, D229A substitution or the triple-mutated L173S,L175S,R176A BamB protein were immunoprecipitated with (+) or without (−) polyclonal anti-BamB antibodies. Immunoprecipitated proteins were separated by SDS-PAGE, transferred onto a nitrocellulose membrane and the presence of BamB or BamA in the samples was visualized by western-blots using polyclonal anti-BamB or anti-BamA antibodies. Hc: immunoglobulin G heavy chain. These results are representative of three independent experiments.

Previous mutational and biochemical studies have shown that D227A or D229A single substitutions of the residue or the simultaneous L173S, L175S, R176A substitutions of the residues in BamB, produce a defective interaction with BamA in *E. coli*. By contrast, single L173S or R176A substitution only weakens the interaction between BamA and BamB in this bacterium [16]. In order to determine whether these residues are involved in the interaction of BamB with BamA in *S.* Enteritidis, recombinant plasmids encoding wild-type BamB-His_6_ or BamB-His_6_ variants were introduced in the *S.* Enteritidis LA5Δ*bamB* mutant and co-immunoprecipitation experiments were performed using a BamB antiserum. Under our conditions, we were not able to detect a protein with the molecular mass reported for the BamA protein (≈90 kDa) after SDS-PAGE of the samples and silver-staining of the gel (data not shown). Thus, we performed western-blot analyses on the same samples (Figure 1). The signal obtained for heavy IgG immuno-globulin chains testified an equivalent antibody precipitation and loading for each sample in our experiments. As expected, in the presence of anti-BamB antibodies, the BamB protein was detected in all the samples except for the LA5Δ*bamB* mutant. Moreover, BamA was found to co-immunoprecipitate in the strain expressing the wild-type BamB protein, but not in the Δ*bamB* mutant. When co-immunoprecipitation experiments were performed with the BamB variants harboring the single substitution L173S, R176A or D227A, BamA was detected at a level similar to that observed for the strain expressing the wild-type BamB protein. By contrast, BamA was not detected in the samples corresponding to the strain expressing the D229A or the triple mutated BamB proteins (Figure 1).

Therefore, no involvement of the L173, R176 or D227 residue alone was observed. Only the D229A substitution or the simultaneous L173S, L175S and R176A substitutions in BamB dramatically impaired the BamB/BamA interaction in *Salmonella*.

### Influence of *bamB* point mutations on the susceptibility of *Salmonella* to various antibiotics

BamB has previously been shown to be involved in outer-membrane biogenesis of *E. coli* and *S.* Enteritidis. It has been shown that a Δ*bamB* mutant presents a decrease in β-barrel protein assembly in the outer membrane and increased susceptibility to several antibiotics: vancomycin, rifampin, erythromycin, novobiocin and bacitracin, which belong to various antibiotic families and have different solubilities and molecular sizes [11], [18], [27]. However, *in vivo* experiments in mice and chickens suggest that the susceptibility to antimicrobials of a *Salmonella* Δ*bamB* mutant is not as affected as would be expected. Indeed, after oral inoculation, this mutant was able to persist as well as the wild-type strain in the animal intestine where the bacteria encounter many molecules with antimicrobial properties (unpublished results). Thus, we first investigated the susceptibility of the *S.* Enteritidis LA5Δ*bamB* to a larger panel of antibiotics belonging to the main antibiotic families, using a disk diffusion assay. We found that the deletion of *bamB* clearly had no impact on the resistance level of *Salmonella* to most of the antibiotics tested (Table S1). The Δ*bamB* mutant of *Salmonella* was more susceptible than the wild-type strain to only vancomycin, rifampin, erythromycin and bacitracin as previously described, and to a lesser extent to two fluoroquinolones: flumequin and enrofloxacin. Interestingly, the susceptibility of the mutant was not related to the molecular size, the solubility or the family of the molecules tested. In a second step, we investigated the roles of the BamB L173, L175, R176, D227, D229 residues in the susceptibility of the LA5Δ*bamB* mutant, expressing a wild-type or a mutated BamB protein in trans, to these six antibiotics (Table 1). Amoxicillin was also added to demonstrate the presence of the recombinant plasmids in the different strains. L173S and R176A BamB variants were able to restore the susceptibility of the LA5Δ*bamB* mutant to the same level as that of the wild-type BamB protein, whereas the strains expressing the D227A or the triple L173S,L175S,R176A BamB variant were not. Lastly, the D229A BamB variant was shown to confer an intermediate phenotype to the Δ*bamB* mutant. Indeed, while this variant restored the level of susceptibility to vancomycin, erythromycin and bacitracin to that of the wild-type BamB, it did not fully restore the level of susceptibility to rifampin, flumequin or enrofloxacin. Such selective sensitivity to rifampin has previously been described in *E. coli* for a *bamC* mutant [28].

**Table 1:** Impact of *bamB* point mutations on antibiotic susceptibility of *S.* Enteritidis.

	AMX	VAN	ERY	RIF	BAC	UB	ENR
LA5	33	6	9	17	6	14	22
LA5Δ*bamB*	34	12	15	28	14	22	29
wtBamB	6	6	9	17	6	16	25
L173S	6	6	6	17	6	17	25
R176A	6	6	6	18	6	17	25
D227A	6	12	15	26	13	20	27
D229A	6	6	6	21	6	19	25
L173S,L175S,R176A	6	14	15	32	16	25	30

Susceptibility to amoxicillin (AMX), vancomycin (VAN), erythromycin (ERY), rifampin (RIF), bacitracin (BAC), flumequine (UB) and enrofloxacin (ENR) was determined using a disk diffusion assay on *S. enterica* serovar Enteritidis LA5Δ*bamB* mutant strain, and the LA5Δ*bamB* mutant complemented with the wild-type BamB protein (wtBamB) or BamB variants. Inhibition zones are given in mm (disk diameter = 6 mm).

In conclusion, these results suggest that the L173 or R176 residue of BamB alone does not play a major role in the intrinsic level of antibiotic susceptibility. By contrast, the D229 residue is important in this susceptibility, but to a lesser extent than the D227 residue, while the triple L173,L175,R176 residues of BamB are essential.

### Influence of *bamB* point mutations on OMP biogenesis in *Salmonella*

**Figure 2:**
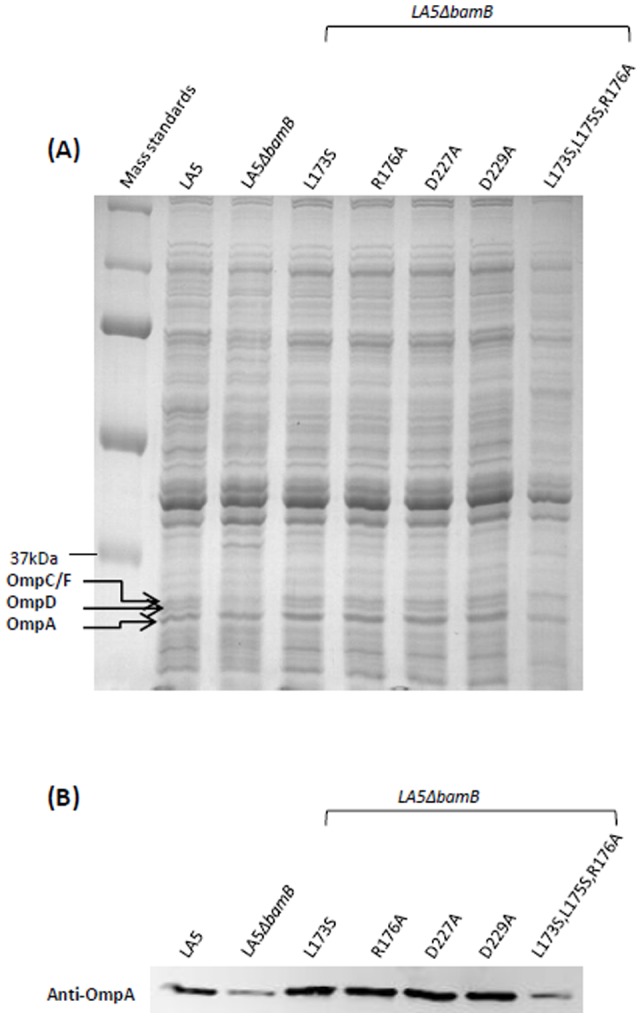
Influence of *bamB* point mutations on outer-membrane protein level. After culture in LB broth, total membrane proteins of the *S.* Enteritidis wild-type strain LA5, the LA5Δ*bamB* mutant, or the LA5Δ*bamB* mutant expressing BamB variants were extracted. Membrane proteins were analyzed by 12% SDS-PAGE and either stained with colloidal Coomassie brilliant blue G250 (A) or transferred onto a nitrocellulose membrane and probed with an anti-OmpA serum (B). The positions of OmpC/F, OmpD and OmpA are indicated. Results shown are representative of at least three independent culture and protein extractions.

In order to observe the influence of the different amino-acid substitutions in BamB on β-barrel protein assembly, the membrane protein profile of our strains was analyzed by SDS-PAGE and western-blot using an anti-OmpA serum. As expected, the LA5Δ*bamB* mutant showed a lower amount of β-barrel proteins such as OmpA, OmpC/F and OmpD than its parental strain (Figure 2A and 2B). Similar results were obtained for the strain producing the L173S,L175S,R176A BamB variant for which a decrease in all these major porins and in the OmpA level was noticed (Figure 2). Surprisingly, we observed that the Δ*bamB* strains producing the BamB variants with single residue substitution had wild-type level of OMP in their respective outer-membranes. This included the D227A variant which was not able to restore the antibiotic susceptibility of the Δ*bamB* mutant and the D229A variant whose interaction with BamA was greatly impaired. Indeed, compared to the wild-type strain, these strains showed the same amount of OmpC/F, OmpD and OmpA on the Coomassie Brilliant blue stained gel and the same amount of OmpA on the anti-OmpA western-blot (Figure 2). These data clearly show that the alteration of the OMP assembly in a Δ*bamB* strain can be complemented by the expression of all our BamB variants except the L173S,L175S,R176A BamB protein.

### Influence of *bamB* point mutations on the expression of T3SS-1 and flagella

**Figure 3:**
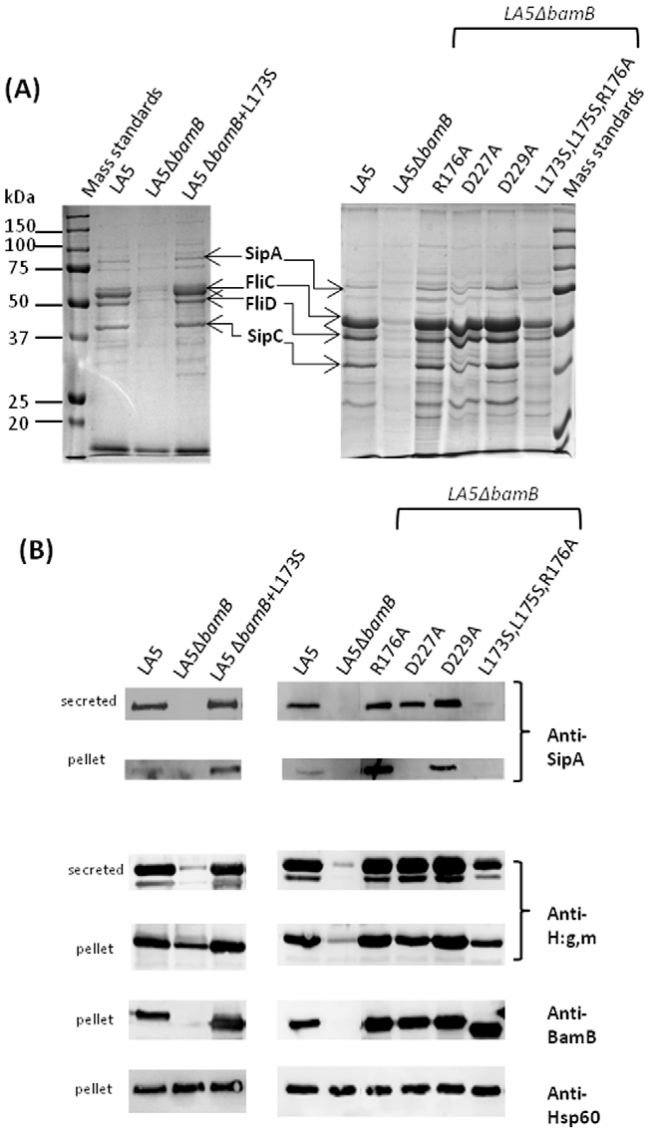
Influence of *bamB* point mutations on T3SS-1 and flagella expression and functionality. Protein secretion by the *S.* Enteritidis wild-type strain LA5, the LA5Δ*bamB* mutant, or the LA5Δ*bamB* mutant expressing BamB variants grown in LB supplemented with 0.3M NaCl was analyzed. Proteins from culture supernatants were TCA precipitated, separated by 10% SDS-PAGE, and stained with colloidal Coomassie brilliant blue G250 (A) or transferred onto a nitrocellulose membrane and probed with polyclonal antibodies raised against either SipA or H:g,m flagellin (B). From the same cultures, bacteria were pelleted and resuspended in Laemmli buffer. After SDS-PAGE and transfer of the samples onto a nitrocellulose membrane, western-blots were performed using anti-SipA, anti-H:g,m flagellin, anti-Hsp60 or anti-BamB sera (B).

Genes related to T3SS-1 and flagella biosynthesis are transcribed less in a *Salmonella* Δ*bamB* mutant than in the wild-type strain. Consequently, the expression of T3SS-1 effectors and flagellar proteins and their secretion in culture supernatant are reduced [18]. In order to determine whether the BamB variants were able to complement a Δ*bamB* mutant, i.e. to restore its ability to synthesize and assemble functional T3SS-1 and flagella, we investigated the expression and secretion of T3SS-1 effectors and flagellar proteins in the pellet and in the supernatant from each bacterial culture grown in LB medium containing NaCl 0.3 M. This culture condition is known to favor the expression of T3SS-1 related genes and to allow flagellar gene expression [29]. To ensure that precipitation of the secreted proteins and the loading of the different samples on the gel were similar, β-lactoglobulin was added to each culture supernatant. In the pellet samples, loading was checked by detecting the constitutively expressed cytoplasmic protein Hsp60. In addition and as expected, a western-blot against BamB confirmed the correct expression of BamB and its variant in all the strains except the deletion mutant (Figure 3A and 3B). Consistent with our previous results, the LA5Δ*bamB* mutant expressed and secreted less SipA and SipC, which are two T3SS-1 effectors, and less FliC and FliD flagellar proteins than the LA5 strain. Furthermore, single amino-acid BamB variants L173S, R176A or D229A were able to completely restore the expression and secretion abilities of the Δ*bamB* mutant. The corresponding strains were all able to express and secrete SipA, SipC, FliC and FliD proteins at similar or even higher levels than those observed with the wild-type strain (Figure 3A and 3B). The last single amino-acid variant, i.e. the D227A BamB variant, was shown to restore the secretion ability of the Δ*bamB* mutant to the wild-type level. However, a lower level of SipA, and to a lesser extent of FliC, were detected by western-blot on the pellet sample of the mutant expressing D227A BamB compared to the other strains which express the same level of BamB variants. As the total amount of these proteins synthesized by the bacteria corresponds to the amount of proteins in the supernatant plus those in the pellet, these results may reflect a slightly lower expression of T3SS-related proteins in the Δ*bamB* mutant expressing BamB D227A than in the wild-type strain, which is, however, sufficient to fully restore T3SS ability to secrete the synthesized effectors. By contrast, the levels of these proteins in the supernatant and pellet greatly decreased for the sample corresponding to the Δ*bamB* mutant expressing the L173S,L175S,R176A BamB protein, thus demonstrating that this protein is not able to complement the Δ*bamB* mutant (Figure 3A and 3B).

Overall, these results demonstrate that the single amino-acid substitutions L173S, R176A, D227A or D229A in BamB have little or no influence on the role of this protein in flagellar expression, or T3SS-1 expression or functionality. By contrast, the simultaneous substitution of the residues L173,L175,R176 plays a crucial part in this control of T3SS expression.

### Influence of *bamB* point mutations on the virulence of *Salmonella* in mice

As the BamB variants displayed different phenotypes *in vitro*, we focused our study on the virulence of the Δ*bamB* mutant expressing these BamB variants in order to understand the relative importance of each phenotype in the strong virulence defect of the Δ*bamB* mutant in mice [18]. For this purpose, BamB variants were expressed from the pACYC177 vector after cloning the corresponding *bamB* ORF under the control of the *aph* constitutive promoter present on this plasmid. This was performed to ensure correct expression of BamB proteins in mice. The pBAD24 vector was not used here since arabinose induction cannot occur *in vivo* as this sugar is not naturally present in mice. It is important to note that the *in vitro* phenotypes of the Δ*bamB* mutants harboring the different recombinant pAC*bamB* plasmids were shown to be identical to those obtained with Δ*bamB* mutants expressing BamB variants from pBAD24 (data not shown).

**Figure 4:**
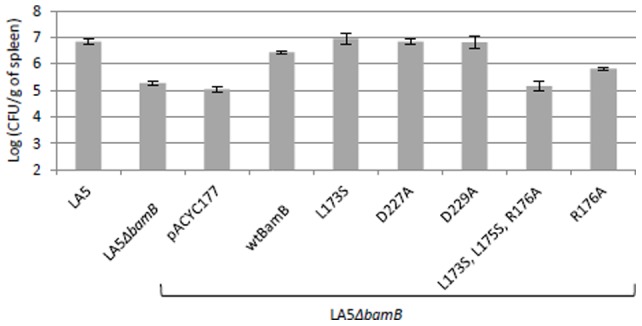
Influence of *bamB* point mutations on *Salmonella* virulence in mice. Groups of 10 six-week-old BALB/C mice were orally inoculated with about 5.10^8^ CFU of the different *S.* Enteritidis strains. Spleen colonization was determined at six days post-inoculation. At least two independent experiments were performed for each strain. Results corresponding to the compilation of all experiments are shown. Results were compared by variance analysis followed by a Tukey-Kramer test (Systat 13, Systat software). Significant differences are presented in Table S2.

BALB/c mice were inoculated by the oral route with the different *S.* Enteritidis strains and the spleen colonization level was determined (Figure 4). Significant differences are presented in Table S2. At six days postinoculation, the LA5Δ*bamB* mutant and the mutant harboring the empty pACYC177 vector colonized the mouse spleens significantly less than the wild-type or the deletion mutant complemented with the wild-type *bamB* gene (*P<*0.0001), as previously described (Figure 4) [18]. The strains expressing the L173S, D227A or D229A BamB variant were able to restore the virulence of the LA5Δ*bamB* mutant completely, which was not the case for the R176A or the L173S,L175S,R176A triple-mutated protein. The Δ*bamB* mutant, expressing either L173S, D227A or D229A BamB protein, was indeed able to colonize the spleens as the wild-type strain and the Δ*bamB* mutant complemented with the wild-type BamB protein (Figure 4). By contrast, the spleen colonization levels were greatly reduced for mice inoculated with the strain expressing BamB proteins with either the triple L173S,L175S,R176A or the single R176A mutation compared to the wild-type controls (*P<*0.0001). Interestingly, while the triple mutated BamB protein was unable to complement the Δ*bamB* mutant, the strain expressing the R176A protein colonized reproducibly and significantly more mouse spleens than the Δ*bamB* mutant (*P<*0.0001). This result indicates that the R176A BamB protein is able to restore partially the virulence of the Δ*bamB* mutant (Figure 4).

These data clearly show that the association of the three residues L173,L175,R176 is crucial for the role of BamB in the virulence of *Salmonella* and that the R176 residue is important for this phenotype. By contrast, no involvement of the L173, D227 or D229 residue of BamB alone was observed.

## Discussion

**Figure 5:**
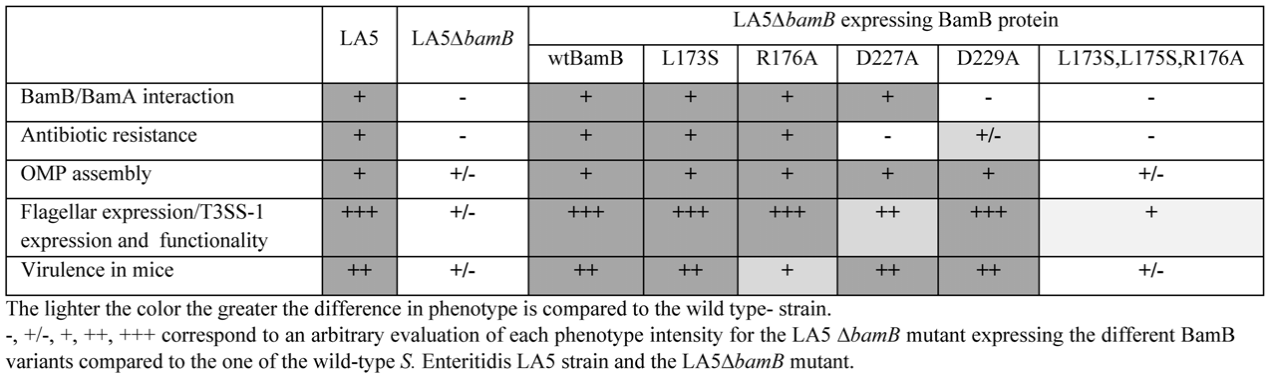
Overview of phenotypic traits of S. Enteritidis LA5ΔbamB mutant strains expressing BamB proteins with point mutations.

In the literature, the BamB protein has been shown to interact with BamA in the BAM complex and to be involved in four different phenotypes. Both in *E. coli* and *Salmonella,* BamB has been shown to be involved in β-barrel protein assembly in the outer membrane and in the preservation of the outer membrane permeability to antibiotics [3], [11], [18], [28]. In *Salmonella,* BamB is also important for optimal expression of the T3SS-1 and flagella related proteins and for bacterial virulence [18], [20]. However, to date little was known about whether an interaction between BamB and BamA is required for BamB activity or the relationships between the different BamB-related phenotypes. Figure 5 summarizes the results obtained in this study.

In *E. coli,* a previous study by Vuong *et al.* [16] looked at the impact of the L173S, L175S, R176A, D227A, D229A single substitutions or the triple L173S,L175S,R176A substitutions in BamB. They analyzed the interaction of this protein with BamA and the ability of these mutated BamB proteins to restore the susceptibility of a *bamB* mutant to vancomycin and its defect in the assembly of the OMP LamB. Most of the results obtained in *Salmonella* are in line with those obtained in *E. coli*. Indeed, the L173S or R176A BamB variants were shown to behave as the wild-type BamB protein, while the triple-mutated BamB variant was not able to complement a *bamB* mutant for the phenotypes analyzed in either of these two bacteria. For example, western-blot analyses of total LamB proteins in *E. coli* [16] and of the amount of OmpA proteins on total *Salmonella* membrane proteins (Figure 2), showed in both cases an important defect in OMPs in the triple-mutated L173S,L175S,R176A BamB variant. Thus, our study confirms the importance of the association of L173, L175 and R176 amino-acids for BamB functions. Indeed, this variant was the only one unable to restore any of the phenotypes tested, including, for the first time, bacterial virulence. These three residues are present in the interconnecting loop 4 that connects blade 4 and 5 of the β-propeller and which seems important for BamB interaction with the POTRA 3 domain of BamA [26]. However, this involvement in the interaction with BamA is probably not the reason why these residues are so important for all BamB functions. In fact, other results of our study strongly suggest that this interaction is not necessary for at least some BamB functions (see below).

However, our study highlights two differences between *E. coli* and *Salmonella*. Firstly, in *Salmonella*, only D229A or the simultaneous L173S, L175S and R176A substitutions induced a marked alteration of BamB interaction with BamA (Figure 1), whereas in *E. coli* the D227A substitution also had an important effect on this interaction [16]. In our experiments, the D227A BamB variant was able to co-immunoprecipitate BamA at levels similar to that obtained for the wild-type BamB protein (Figure 1). The major difference between the procedures used in these two studies is the use of an anti-His tag serum in the study of Vuong *et al.* [16] and of a BamB antiserum in the present study. This result demonstrates that the residue D227 is not important for BamB interaction with BamA in *Salmonella*. The second difference concerns the ability of the BamB D227A variant to restore or not a wild-type level of antibiotic susceptibility in a *bamB* mutant. In *E. coli,* the *bamB* mutant expressing the D227A BamB variant had an intermediate minimal inhibitory concentration of vancomycin situated between that of the mutant and the wild-type strain. By contrast, in our experiments performed with *Salmonella*, no complementation was observed with the D227A variant of BamB for the six antibiotics tested, including vancomycin (Table 1). Recall that the plasmids used in our study are those of Vuong *et al.* [16] and thus the cloned *bamB* gene used for complementation analyses is that of *E. coli*. We therefore can not exclude that the two differences between *E. coli* and *Salmonella* we observed could be due to the fact that we did not use the *bamB* gene of *Salmonella* even if the two BamB proteins are very similar (94% of similarity and 91% of identity) and the amino-acids studied are conserved in the two bacteria.

In the literature, the BamB protein has until now been considered as a “permanent” member of the BAM complex, interacting only with the BamA protein in this complex [3], [9]. Our results suggest that the interaction of BamB with BamA is not absolutely necessary for correct OMP biogenesis, optimal T3SS expression and virulence in mice, and therefore that BamB could be active “outside” the BAM complex. Indeed, while the D229A BamB variant was shown to restore a wild-type level of OMP in the outer membrane, T3SS-1/flagella expression and virulence in mice, it was unable to interact with BamA (Figures 2 and 3). Vuong *et al.* also previously found that this mutation did not affect the LamB level and the minimal inhibitory concentration of vancomycin [16]. However, we cannot exclude the hypothesis that a weak interaction of BamB with BamA in vivo could be sufficient to correctly assemble OMPs in the outer membrane, fully express T3SS and allow *Salmonella* to be fully virulent. If the BamB/BamA interaction is not absolutely required for BamB activity, it could explain the results of Ieva *et al.* [19] suggesting a direct role of BamB in β-barrel assembly at a later stage than BamA.

Overall, our results invoke a possibility that BamB can function in OMP assembly both in conjunction with the BAM complex and independent of the BAM complex. This hypothesis could explain why *bamB* and *surA* mutants have similar phenotypes [30]. Moreover, our results show that the antibiotic sensitivity phenotype of a Δ*bamB* mutant is not always associated with an OMP assembly defect. We conclude this from the observation that the BamB D227A and to a lesser extent the D229A BamB variants restore a wild-type level of the major OMP, but not of antibiotic susceptibility to a Δ*bamB* mutant.

BamB plays an essential role in *Salmonella* virulence in a lethal systemic infection model in mice, but the complete underlying mechanism remains to be elucidated. The attenuation of the virulence of a *Salmonella* Δ*bamB* mutant could be explained by an increase in outer-membrane permeability, making this mutant more susceptible to stomach acidity and to antimicrobial peptides, and/or decreased expression of T3SS and flagella [18], [20]. From our results, it appears unlikely that the susceptibility of the mutant to antimicrobials can explain its virulence defect. While the Δ*bamB* mutant expressing the D227A BamB variant presents an alteration in outer-membrane permeability similar to the mutant, it remains as virulent as the wild-type *S.* Enteritidis LA5 strain. These data are in line with unpublished results from our laboratory which show that the Δ*bamB* mutant is able to persist as well as the wild-type strain in the caeca for several weeks after oral inoculation of chicks. By contrast, it is more than likely that the decreased expression of T3SS in the Δ*bamB* mutant plays an important role in the virulence defect of this strain *in vivo*. Indeed, compelling evidence highlights the major role of T3SS in the virulence of *Salmonella* [31], [32], [33]. However, currently no data from the present study support the *in vivo* importance of BamB in the control of T3SS expression. Indeed, the only BamB variant (L173S,L175S,R176A) impacting on the expression of T3SS-1 and flagella was also impaired in all the *in vitro* and *in vivo* phenotypes that were tested. Nevertheless, our results obtained in mice with the R176A BamB variant show that the virulence defect of a Δ*bamB* mutant could be linked not only to decreased expression of its T3SS, and thus suggest another role of BamB *in vivo*. The Δ*bamB* mutant expressing this mutated R176A protein, while expressing functional T3SS-1 and flagella, was clearly less virulent than the wild-type strain even though it colonized spleens significantly more than the Δ*bamB* mutant. According to van der Straaten *et al.* [34] and van Diepen *et al.* [35], a role of BamB (called SspJ in their paper) in bacterial resistance to oxidative stress could explain the *in vivo* attenuation that we observed for the Δ*bamB* mutant expressing R176A BamB.

Overall, our results show that in *Salmonella* the interaction of BamB with BamA is not absolutely required for most phenotypes in which BamB has previously been shown to be involved. Moreover, we have demonstrated that some of these phenotypes are independent, thus highlighting that the role of BamB is more complex than previously thought. Further functional experiments are required to understand the precise role of this protein, which is an interesting potential therapeutic target due to its role in several essential functions of *Salmonella,* including outer membrane biogenesis and virulence. On the basis of our present results, the association of L173, L175 and R176 amino-acids could be considered as candidate sites for the design of BamB inhibitors.

## Materials and Methods

### Ethics statement

Animal experiments were conducted in strict accordance with French recommendations (number 2001–131 from 4.02.2001 and number 2001–464 from 29.05.2001) and the protocol was approved by the Regional Ethics Committee for Animal Research “Comité Régional d'Ethique pour l′Expérimentation Animale - Centre Limousin”, recognized by the French Ministry for Research and Education (file number 2011/10). All efforts were made to minimize suffering. We fixed a humane endpoint based on the ability of mice to feed and have a normal activity.

During the experiments, mice were monitored at least once a day and their behavior was noted. As soon as one animal in a group showed at least 2 of the following symptoms, they were monitored twice a day: bristly hairs, a round-shouldered back or conjunctivitis-like symptoms. At the end of the experiment, mice showed the above symptoms but were all still able to feed and had a normal activity. Thus, the humane endpoint was not reached.

Carbone dioxide anesthesia was performed before mice euthanasia using cervical dislocation by trained individuals.

### Bacterial strains and culture conditions

The bacterial strains and plasmids used in this study are presented in Table 2. The *S.* Enteritidis LA5 wild-type strain is a field isolate from infected chickens and is resistant to nalidixic acid (NAL): 20 µg.mL^−1^ [36], [37]. Bacteria were routinely grown in Luria-Bertani (LB) broth with shaking (200 rpm) at 37°C overnight, except when specified. Carbenicillin (CAR): 100 µg.mL^−1^ or NAL: 20 µg.mL^−1^ were added to the culture medium when necessary. In order to induce BamB expression from pBAD24 or pTrcHisA plasmids, arabinose (0.2% [wt/vol]) or isopropyl β-D-1-thiogalactopyranoside (IPTG 1 mM) was added respectively to the medium. The strains expressing a wild-type BamB and those expressing BamB with point mutations had similar growth curves under all culture conditions used in this study (data not shown).

**Table 2:** Bacterial strains and plasmids used in this study.

Strain or Plasmid	Relevant characteristic(s)	Source
Strains		
LA5	*S. enterica* serovar Enteritidis wild-type strain (NAL^R^)	[36]
LA5Δ*bamB*	LA5 isogenic mutant with the *bamB* gene deleted (NAL^R^)	[18]
Plasmids		
pACYC177	Cloning vector (CAR^R^,KAN^R^)	[38]
pTrcHis*A*	Expression vector (CAR^R^) for His-tagged proteins	Invitrogen
pTrcHis*bamB*	*S.* Enteritidis *bamB* gene in plasmid pTrcHis*A* (CAR^R^) producing wtBamB	This work
pBAD*bamB*(L173S)	Plasmid pBAD24 producing L173S BamB variant	[16]
pBAD*bamB*(R176A)	Plasmid pBAD24 producing R176A BamB variant	[16]
pBAD*bamB*(D227A)	Plasmid pBAD24 producing D227A BamB variant	[16]
pBAD*bamB*(D229A)	Plasmid pBAD24 producing D229A BamB variant	[16]
pBAD*bamB*(L173S,L175S,R176A)	Plasmid pBAD24 producing L173S,L175S,R176A BamB variant	[16]
pAC*bamB*	*S.* Enteritidis *bamB* gene in plasmid pACYC177 (CAR^R^) producing wtBamB. Formerly pAC*yfgL*	[18]
pAC*bamB*(L173S)	Plasmid pACYC177 producing L173S BamB variant	This work
pAC*bamB*(R176A)	Plasmid pACYC177 producing R176A BamB variant	This work
pAC*bamB*(D227A)	Plasmid pACYC177 producing D227A BamB variant	This work
pAC*bamB*(D229A)	Plasmid pACYC177 producing D229A BamB variant	This work
pAC*bamB*(L173S,L175S,R176A)	Plasmid pACYC177 producing L173S,L175S,R176A BamB variant	This work

### Construction of plasmids pTrcHis*bamB* and pAC*bamB* mutants

The wild-type *bamB* ORF from the chromosomal DNA of *S.* Enteritidis LA5 strain was amplified by PCR, using primers 5′GCGAATTCCAGGAAAACGGCCCCTACACCAGGAGC3′ and 5′ATCGAGCTCCAATTGCGTAAATTACTTCTGCCAGGG 3′. After restriction with the EcoRI and SacI restriction enzymes, the 1.2 kb PCR product was cloned into the same restriction sites of pTrcHisA vector (Invitrogen), generating pTrcHis*bamB*.

Using PCR, the different mutated *bamB* ORFs (coding for BamB variants with single L173S, R176A, D227A, D229A mutations or the triple L173S,L175S, R176A substitutions) were amplified from pBAD*bamB* plasmids with primers 5′TTGTACCCGGGCGAGAGGGACCCGATG3′ and 5′TAGAAGCTTAGTGATGGTGATGGTGATG3′. After restriction with the SmaI and HindIII restriction enzymes, the 1.2 kb PCR product was cloned into the same restriction sites of pACYC177 vector [38], generating plasmids pAC*bamB* expressing constitutively the different BamB variant proteins thanks to the promoter of the kanamycin resistance gene.

All constructions were checked by sequencing.

### Co-immunoprecipitation

Co-immunoprecipitation assays were performed in accordance with the protocol of Vuong *et al.* [16] with the following changes: cultures were made in LB medium and 40 mL of bacteria were pelleted and resuspended in 300 µl of lysis buffer (Tris-HCl pH 8, 50 mM; NaCl 100 mM, EDTA 0.1 mM, Triton 0.1%, lysozyme 0.35 µM). After protein solubilization and centrifugation, the assays were performed with 11 mg of proteins for each strain using a polyclonal anti-BamB serum and sepharose beads coupled with protein G (Sigma-Aldrich) for immunoprecipitation. Five washes were performed on SigmaPrep spin columns (Sigma-Aldrich) with a solution containing 500 µl of a Tris-HCl 50 mM pH 8; NaCl 350 mM; EDTA 0.1 mM. Immunoprecipitates were eluted from columns by boiling for five minutes after the addition of 50 µL of Laemmli buffer [39]. Eluates were separated on a 10% SDS-polyacrylamide gel. Proteins were then either silver stained or transferred onto nitrocellulose membranes (Protran) and analyzed by western-blots with anti-BamA or anti-BamB antisera as described below. Three independent experiments were carried out for each strain.

### Protein extractions

For secreted proteins and proteins from the pellet, bacteria were cultured in LB broth containing 0.3 M NaCl until the optical density at 600 nm (O.D._600_) reached 1.8–2.0. Secreted bacterial proteins were then recovered as previously described by Arricau *et al*. [29]. β-lactoglobulin (0.5 µg.mL^−1^; Sigma) was added to each culture supernatant to control for protein precipitation and sample loading. Bacterial pellet proteins were obtained after centrifugation of bacterial cultures and direct resuspension in Laemmli buffer [39]. Proteins were then separated using electrophoresis in 10% SDS-polyacrylamide gels and either stained with colloidal Coomassie brilliant blue G-250 [40] or transferred onto nitrocellulose membranes (Protran) for western-blotting. At least three independent experiments were carried out.

To recover membrane proteins, bacteria were grown in LB until O.D._600_ = 1 and total membrane protein extractions were performed as previously described [27]. Proteins then underwent electrophoresis on a 12% SDS-PAGE and were stained with colloïdal Coomassie brilliant blue G-250 [40]. At least three independent experiments were carried out for each strain.

### Western-blot analyses

Proteins were analyzed through western-blots using, as primary antibodies, either a polyclonal rabbit anti-SipA serum (1∶2000) [20], a polyclonal rabbit anti-H:g,m serum (1∶500) (Biolabs), a polyclonal mouse anti-Hsp60 serum (1∶6000) (Assaydesigns-Stressgen), a polyclonal rabbit anti-BamA serum (1∶40000) [41], a polyclonal rabbit anti-BamB serum (1∶6000) or a polyclonal rabbit anti-OmpA serum (1∶6000). The anti-H:g,m serum recognizes flagellar proteins including FliC and FliD proteins. A goat peroxydase-labelled anti-rabbit IgG (1∶10000, Dako) serum was used as secondary antibody except for Hsp60, for which a rabbit peroxidase-labelled anti-mouse serum (1∶5000, Dako) was used. Proteins were revealed using the SuperSignal West Dura Extended Duration Substrate (Thermo Scientific).

### Antibiotic susceptibility

Strain susceptibilities to different antibiotics were assessed by a disk diffusion assay using 6-mm filter paper disks (Bio-Rad) as recommended by the European Committee on Antimicrobial Susceptibility Testing. From overnight cultures, bacterial suspensions were prepared at the 0.5 McFarland turbidimetric standard. They were then poured over a Mueller-Hinton (MH) agar plate containing arabinose (0.2% [wt/vol]) or IPTG (1 mM) when needed, and after drying, 6 mm disks containing the antibiotics were placed on the agar surface. After plate incubation for 20 h at 37°C, the diameter of the growth inhibition zone around each disk was measured. Two experiments were carried out for each strain. We checked that the addition of arabinose or IPTG in the pre-culture and in the MH agar plates had no impact on the diameter obtained for the wild-type or the *bamB* mutant strains (data not shown).

### Infection of mice

Six to seven week-old female BALB/c mice were obtained from Janvier laboratories and maintained in our animal facilities at the Institut National de la Recherche Agronomique (Nouzilly, France) on a diet of mouse chow and water ad libitum. Groups of 10 mice were each inoculated orally with approximately 5.10^8^ CFU of *S.* Enteritidis using the protocol described by Pardon *et al.* [42]. Spleen colonization was estimated at six days postinoculation by plating serial dilutions in phosphate-buffered saline on *Salmonella-Shigella* agar plates. Three independent experiments were carried out. Results were compared using analysis of variance and analyzed by the Tukey-Kramer test (Systat 13, Systat software).

## Supporting Information

Table S1 **Impact of *bamB* deletion on the antibiotic susceptibility of *S*. Enteritidis.**
(DOC)

Table S2 **Pairwise comparison of spleen colonization level by the different *S.* Enteritidis strains.**
(DOC)
