# Supplementary material for: Deciphering the Roles of BamB and Its Interaction with BamA in Outer Membrane Biogenesis, T3SS Expression and Virulence in Salmonella
Source: PLoS One. 2012 Nov 5;7(11):e46050. doi: 10.1371/journal.pone.0046050 (PMC3489874; doi:10.1371/journal.pone.0046050)
Supplement: Table S1 — Impact of bamB deletion on the antibiotic susceptibility of S. Enteritidis. (DOC) [file pone.0046050.s001.doc]

**Table S1. Impact of *bamB* deletion on the antibiotic susceptibility of *S*. Enteritidis.**

|  | AMX | VAN | ERY | RIF | BAC | UB | ENR | SSS | CRO | AN | TE | SPT | IPM | FEP | TEC | CHL | TMP | CIP | FFC |
| --- | --- | --- | --- | --- | --- | --- | --- | --- | --- | --- | --- | --- | --- | --- | --- | --- | --- | --- | --- |
| LA5 | 30 | 6 | 9 | 16 | 6 | 16 | 23 | 29 | 32 | 24 | 25 | 20 | 34 | 34 | 6 | 27 | 29 | 27 | 25 |
| LA5Δ*bamB* | 32 | 14 | 14 | 28 | 14 | 22 | 28 | 28 | 34 | 24 | 26 | 21 | 34 | 34 | 7 | 30 | 34 | 29 | 28 |

Susceptibility to amoxicillin (AMX), vancomycin (VAN), erythromycin (ERY), rifampin (RIF), bacitracin (BAC), flumequine (UB), enrofloxacin (ENR), sulfamids (SSS), ceftriaxone (CRO), amykacin (AN), tetracyclin (TE), spectinomycin (SPT), imipenem (IPM), cefepim (FEP), chloramphenicol (CHL), trimethoprim (TMP), ciprofloxacin (CIP) and florphenicol (FFC) was determined using a disk diffusion assay on *S.* Enteritidis LA5 and LA5∆*bamB* strains. Inhibition zones are given in mm (disk diameter=6mm).
