# Supplementary material for: Deciphering the Roles of BamB and Its Interaction with BamA in Outer Membrane Biogenesis, T3SS Expression and Virulence in Salmonella
Source: PLoS One. 2012 Nov 5;7(11):e46050. doi: 10.1371/journal.pone.0046050 (PMC3489874; doi:10.1371/journal.pone.0046050)
Supplement: Table S2 — Pairwise comparison of spleen colonization level by the different S. Enteritidis strains. (DOC) [file pone.0046050.s002.doc]

**Table S2. Pairwise comparison of spleen colonization level by the different *S.* Enteritidis strains.**

| **Strain 1** | **Strain 2** | ***P* (**≤**)** |
| --- | --- | --- |
| LA5 | LA5Δ*bamB* | 0.0001 |
| LA5 | pACYC177 | 0.0001 |
| LA5 | R176A | 0.0001 |
| LA5 | L173S,L175S,R176A | 0.0001 |
| LA5Δ*bamB* | wtBamB | 0.0001 |
| LA5Δ*bamB* | L173S | 0.0001 |
| LA5Δ*bamB* | R176A | 0.0001 |
| LA5Δ*bamB* | D227A | 0.0001 |
| LA5Δ*bamB* | D229A | 0.0001 |
| wtBamB | pACYC177 | 0.0001 |
| L173S | pACYC177 | 0.0001 |
| L173S | R176A | 0.0001 |
| L173S | L173S,L175S,R176A | 0.0001 |
| R176A | pACYC177 | 0.0001 |
| R176A | wtBamB | 0.0001 |
| D227A | pACYC177 | 0.0001 |
| D227A | wtBamB | 0.019 |
| D227A | R176A | 0.0001 |
| D227A | L173S,L175S,R176A | 0.0001 |
| D229A | pACYC177 | 0.0001 |
| D229A | R176A | 0.0001 |
| D229A | L173S,L175S,R176A | 0.0001 |
| L173S,L175S,R176A | wtBamB | 0.0001 |
| L173S,L175S,R176A | R176A | 0.005 |

P-values (P) obtained after a Tukey-Kramer test (Systat 13, Systat software) performed on the in vivo data presented on Figure 4. Only significant p-values are given.
